# Construction of a predictive model for cognitive impairment among older adults in Northwest China

**DOI:** 10.3389/fnagi.2025.1487838

**Published:** 2025-07-31

**Authors:** Yu Wang, Ni Wang, Yanjie Zhao, Xiaoyan Wang, Yuqin Nie, Liping Ding

**Affiliations:** ^1^Zhejiang Provincial People’s Hospital, Hangzhou, Zhejiang, China; ^2^School of Nursing, Xinjiang Medical University, Ürümqi, Xinjiang, China; ^3^The Second Affiliated Hospital of Xinjiang Medical University, Ürümqi, Xinjiang, China

**Keywords:** cognitive impairment, older adults in Northwest China, random forest algorithm, multivariate logistic regression model, predictive model

## Abstract

**Background:**

Cognitive impairment is most common in older adults and seriously affects their quality of life. Early prediction of cognitive impairment could be beneficial for identifying vulnerable individuals and planning primary and secondary prevention to reduce the incidence of cognitive impairment. The aim of this study is to combine the advantages of machine learning and logistic regression to construct a risk prediction model for cognitive impairment among older adults in Northwest China to identify individuals at increased risk.

**Methods:**

A cross-sectional study was conducted. The participants and data included in this study were from the National Key Research and Development Project “Intelligent Elderly Disability Monitoring and Early Warning Network System Construction.” Older adults in Northwest China were assessed between March 2022 and January 2023 using a multistage sampling method. We used random forest algorithms to select important features from potential predictors. The features identified using the random forest model were subjected to logistic regression analysis to develop a cognitive impairment prediction model. Model performance was evaluated on the basis of the area under the curve, sensitivity, specificity, accuracy, F1 score, precision, and recall.

**Results:**

A total of 12,332 older adults were recruited and screened with the Mini-Mental State Examination Scale. The detection rate of cognitive impairment was 24.86%. The random forest algorithm and multifactorial logistic regression analysis revealed that the independent predictive factors for cognitive impairment among older adults in Northwest China were advanced age, high BMI, low literacy, low gait speed, primary financial resources from children or labor, freelance work, less exercise, low scores on instrumental activities of daily living, low walking test scores, low levels of activities of daily living, and irregular participation in social activities, all of which were used to create the nomogram. The model established with the above 12 independent predictors achieved an area under the curve of 0.816 (95% CI: 0.807∼0.824); the risk prediction value of 0.211 was the best cut-off value and showed good sensitivity (75.50%), specificity (72.40%), accuracy (73.14%), F1 score (0.802), precision (89.91%), and recall (72.38%).

**Conclusion:**

The prevalence of cognitive impairment in older adults is high in Northwest China. The combination of machine learning and logistic regression yielded a practical cognitive impairment prediction model and has great public health implications for the early identification and risk assessment of cognitive impairment among older adults in Northwest China.

## 1 Introduction

Cognitive impairment is characterized by a disruption to one or more cognitive functions, such as thinking, memory, language, attention, perception, and executive functions (Lezak, 2004). Cognitive impairment is common in older adults and is associated with adverse outcomes, such as dementia and Alzheimer’s disease (Morley, 2018). There is a high prevalence of cognitive impairment in the elderly population, which is accompanied by many related risk factors, complex etiologies, and great harm (Morley, 2018). Moreover, cognitive decline is the main cause of morbidity and mortality in the global population (Daviglus et al., 2010), and the prevention and treatment of cognitive impairment has become an important public health issue. A statistical analysis of the dementia population revealed that, on average, one person in the world develops dementia every 3 s. China, as the country with the fastest growing elderly population (Man et al., 2021), has more than 68 million older adults with mild cognitive impairment, dementia, or Alzheimer’s disease in 2020 (Jia et al., 2020), representing a heavy disease burden throughout China (Cheng, 2017). The ability to predict the incidence of cognitive impairment is critically relevant for decisions made by health and social services policy-makers to plan and provide intermediate and long-term care services for older adults in China. Early and precise prevention and intervention for high-risk populations, such as those receiving cognitive training, moderately intensive physical exercise and diet, could effectively reduce the risk of dementia and save enormous medical resources from those who are less likely to develop cognitive impairment (Kasper et al., 2020; Wang et al., 2023).

A prediction model may be an efficient method to identify individuals at high risk for cognitive impairment. Numerous cognitive impairment prediction models have been developed in previous studies (Jin et al., 2023; Sakal et al., 2024; Zhang X. et al., 2024; Zhang X. et al., 2024). The predictors incorporated in these models varied. Nearly all existing models make predictions by leveraging measures of cognition, age, and education. Additional covariates vary from model to model and include factors such as activities of daily living (ADL), hobbies such as gardening and watching television, and marital status (Jin et al., 2023; Sakal et al., 2024; Zhang X. et al., 2024; Zhang X. et al., 2024). Differences between predictors of cognitive impairment should be considered more carefully. Furthermore, population characteristics vary widely across China, and risk factors that are the most predictive differ across different socioeconomic groups (Sakal et al., 2024). In addition, no prediction model has primarily tested the population in Northwest China alone.

Numerous studies have demonstrated that machine learning algorithms (such as extreme gradient boosting, gradient boosting decision tree, and random forest) outperform traditional statistical models (such as logistic regression and Cox proportional hazards regression) in terms of predictive performance across medical fields (Goldstein et al., 2017; Kakadiaris et al., 2018; Xi et al., 2022) because of their ability to analyze and learn the complex interactions and non-linear associations among variables (Beam and Kohane, 2018; Schauberger et al., 2023). However, traditional statistical models still have irreplaceable strengths, including their natural transparency, interpretability, and robustness, which increase their practicality in clinical research (van der Ploeg et al., 2014). Therefore, using machine learning algorithms alone or traditional regression methods alone to train prediction models usually results in either accurate but complicated black boxes or practical but unsatisfactory scoring systems.

In this study, we combined machine learning (random forest) with logistic regression to develop a straightforward and practical risk prediction model to help better identify individuals at risk of cognitive impairment among older adults in Northwest China. On the basis of predictors from existing prediction models (Jin et al., 2023; Sakal et al., 2024; Zhang X. et al., 2024; Zhang X. et al., 2024) and data from the National Key Research and Development Project “Intelligent Elderly Disability Monitoring and Early Warning Network System Construction,” which contains participants aged 60 years and older in Northwest China, we establish a prediction model for cognitive impairment. In this study, we examined how well demographic factors (such as sex, age, ethnicity, education level, chronic disease, exercise, smoking, etc.), balance, gait speed, muscle strength and activities of daily living predict cognitive impairment in the older population in Northwest China. These findings provide a scientific basis for the subsequent prevention and development of interventions and are clinically important for identifying the risk of cognitive impairment in elderly people.

## 2 Materials and methods

### 2.1 Study subjects

This study used multistage sampling to analyze baseline data collected between March 2022 and January 2023 from eight tertiary hospitals, nine secondary hospitals, seven community health service centers, and eight elderly care facilities in 4 regions of the Xinjiang Uygur Autonomous Region of China (Wuchang, South Xinjiang, North Xinjiang, and East Xinjiang). These data were obtained from the National Key Research and Development Project “Intelligent Elderly Disability Monitoring and Early Warning Network System Construction,” which recruited participants aged 60 years and older. The inclusion and exclusion criteria for participants are shown in Table 1. The study was performed in accordance with the principles of the Declaration of Helsinki. All patients provided informed consent, and the study was approved by the Ethics Committee of Beijing Hospital (2021BJYYEC-325-01).

**TABLE 1:** The inclusion and exclusion criteria for participants.

Inclusion criteria	Exclusion criteria
Individuals with age ≥ 60 years and good communication skills.	(1) Had schizophrenia or other mental illnesses;
	(2) Were unable to cooperate with the physical function survey (e.g., total disability);
	(3) Were in the acute stage of disease treatment (e.g., undergoing surgery).

### 2.2 Collection of data and quality control

In this study, one assessor was assigned to each site to collect the data. The data were collected using the “Beijing Medical Policy Elderly Functional Assessment Platform” app designed by the research group, and the head of the research group provided uniform training to the heads of the relevant participating units in the form of an online meeting. Prior to data collection, the assessors used unified instructions to explain the purpose and content of the study to the elderly individuals, after which informed consent was obtained before data collection. Moreover, to ensure the quality of the data collection, all the participating researchers received comprehensive training. The inclusion of study subjects was based strictly on the inclusion and exclusion criteria. After data entry, the data were double-checked. The accuracy of the data was confirmed by manual, computerized and logical error assessment of the input information.

### 2.3 Research tools

#### 2.3.1 Basic information questionnaire

We designed a general information questionnaire based on cognitive impairment prediction models developed in previous studies (Jin et al., 2023; Sakal et al., 2024; Zhang X. et al., 2024; Zhang X. et al., 2024). The self-designed general information questionnaire included sex, age, BMI, ethnicity, education level, marital status, type of residence, current employment, type of health insurance, main source of income, long-term medication use, type of chronic disease, alcohol consumption, smoking, annual medical check-ups, whether social activities are held, whether physical activity is performed, and social support.

#### 2.3.2 Mini-Mental State Examination (MMSE)

In 1975, scholars used the MMSE to measure cognitive function, including time and place orientation, immediate memory and recall ability, attention and calculation ability and language ability. The scale has a total score of 30 points, and the higher the score is, the better the cognitive function (Li, 2021). Cognitive impairment is determined by a score of 24 (secondary school education level or above), 20 (primary school education level) or 17 (illiterate), with a 24 h test–retest reliability of 0.89. Wang and Zhang (1989) translated the scale into Chinese in 1989, with a 48–72 h test–retest reliability of 0.91.

#### 2.3.3 Balance test, gait speed and muscle strength assessment

The balance, gait speed and muscle strength of older adults were measured using three dimensions of the Simple Physical Condition Scale developed by the National Center on Aging (National Institute on Aging, 2022). The balance test consisted of two-legged standing, semianterior-posterior standing and anterior-posterior standing, with two-legged combined standing and semianterior-posterior standing > 10 s scoring 1 point, anterior-posterior standing 3∼ < 10 s scoring 1 point, and 10 s scoring 2 points. The gait speed test was a 2.44 m walking speed test, with a score of 1 for < 0.43 m/s, 2 for 0.44–0.60 m/s, 3 for 0.61–0.77 m/s, and 4 for ≥ 0.78 m/s. The muscle strength test was the five-time sit-to-stand test, with 16.70–60 s as 1 point, 13.70–16.69 s as 2 points, 11.20–13.69 s as 3 points, and ≤ 11.19 s as 4 points.

#### 2.3.4 Activities of Daily Living (ADLs)

The ability to perform activities of daily living is determined by assessing basic activities of daily living (BADLs) and instrumental activities of daily living (IADLs). BADLs were evaluated using the Barthel Index (BI), a scale constructed by Mahoney and Barthel (1965) to measure BADL ability; this scale contains 10 items. The scale has a total possible score of 100, with higher scores indicating greater BADL competence. A total score of < 40 was classified as severe dependence, 41–60 was classified as moderate dependence, 61–99 was classified as mild dependence, and 100 was classified as no dependence. IADL ability was measured using the Instrumental Activities of Daily Living (IADLs) Ability Scale, which was constructed by Lawton and Brody (1969) in 1965 to measure IADL ability and included eight items, with a total score ranging from 0 to 24. Higher scores are indicative of greater IADL ability, and a total score of 24 is considered indicative of no IADL limitations.

### 2.4 Statistical analysis

**FIGURE 1:**
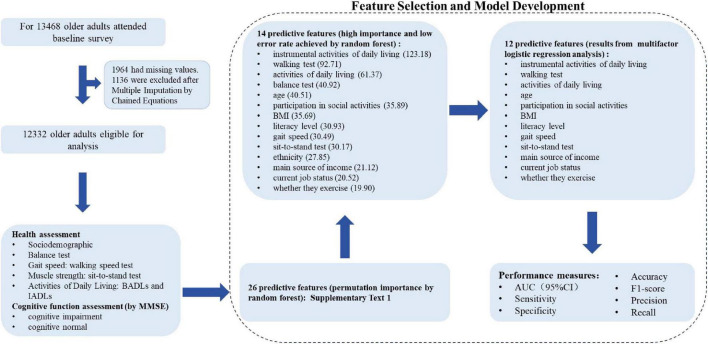
Processes used to build the cognitive impairment diagnostic model.

Statistical analyses were performed using R 4.2.3 software. Normally distributed continuous variables are presented as the means with SDs, and Student’s *t*-test was used for statistical analysis. Non-normally distributed continuous variables are presented as medians and interquartile ranges, and the Wilcoxon rank-sum test was used for comparisons. Categorical variables are presented as counts and percentages and were compared using the chi-square test. The machine learning algorithm was used for feature selection. The top important features with the smallest average out-of-bag error rates selected by the random forest algorithm were used for model development. The random forest algorithm generates multiple decision trees in parallel by conducting random sampling and random feature selection, and the final prediction is made by aggregating the votes from all decision trees. The feature importance in a random forest can be measured by evaluating the mean Gini index of each feature across multiple trees. Logistic regression was used to train the final prediction model using the features selected. The influencing factors with the highest importance scores and the smallest average out-of-bag error rates were subsequently included in the multifactorial logistic regression analysis model. The predictive model was presented as a nomogram, and each variable in the nomogram was assigned a specific score on the rating scale. The predicted probability of cognitive impairment was obtained by summing the scores for each variable and drawing a vertical line down the total score. Model performance was evaluated based on the area under the curve, sensitivity, specificity, accuracy, F1 score, precision, and recall. All the statistical tests were two-sided, and *P* < 0.05 was regarded as statistically significant. The processes of building the cognitive impairment diagnostic model are shown in Figure 1.

## 3 Results and discussion

### 3.1 Results

#### 3.1.1 Description of general information

Among the 12,332 older adults included in this study, 24.86% were diagnosed with cognitive impairment. The differences in all the potential predictors (excluding sex) between participants with and without cognitive impairment were statistically significant (see Supplementary Table 1).

#### 3.1.2 Ranking the importance of factors influencing cognitive functioning

**FIGURE 2:**
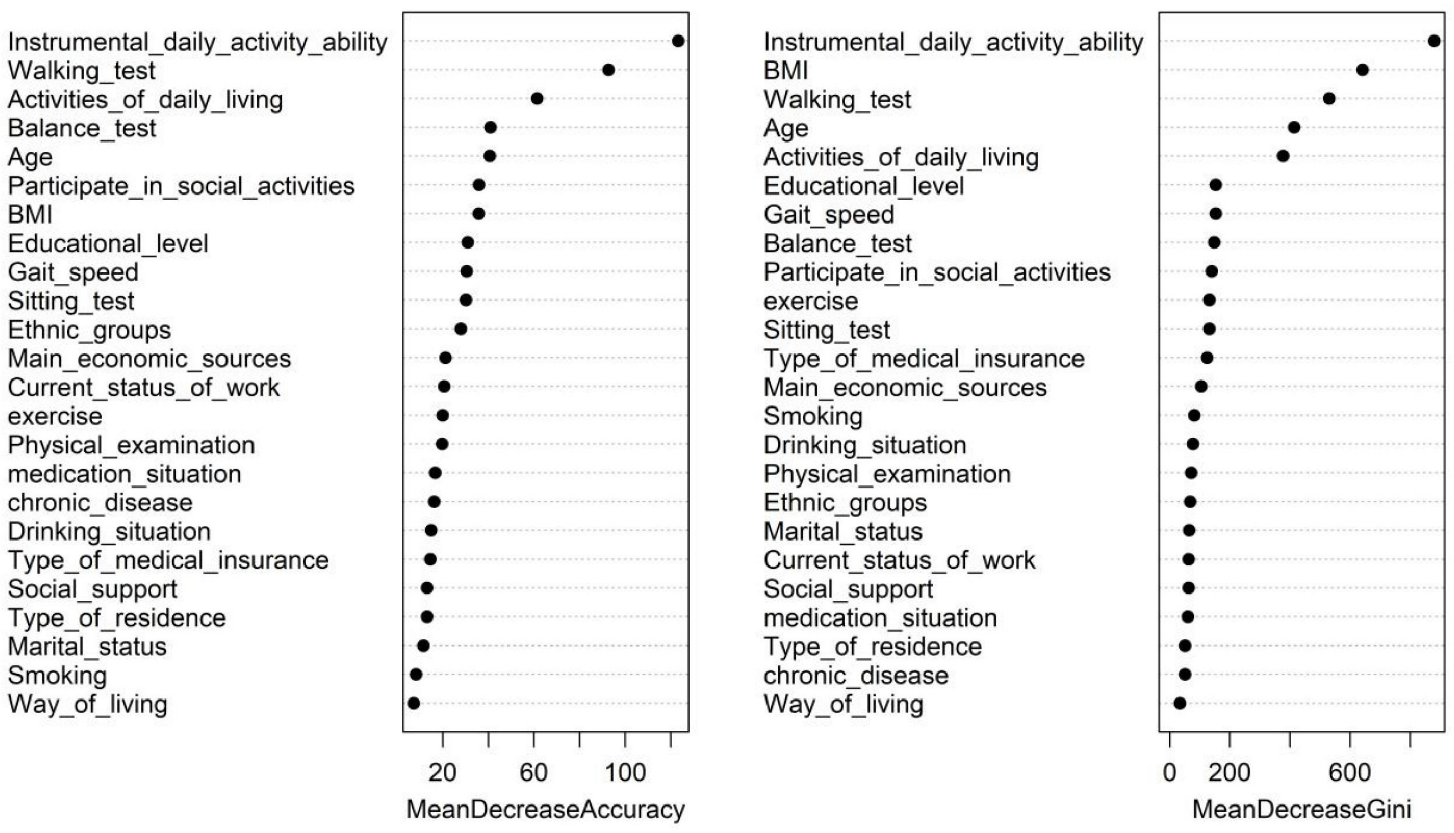
Ranking of the importance of factors influencing cognitive impairment in old age.

Using the occurrence of cognitive impairment as the dependent variable, 26 influencing factors involving general demographics, self-care in daily living, the Mini-Mental State Examination score, activities of daily living, balance, gait speed and muscle strength were ranked in order of importance using the random forest algorithm. The results revealed that the MDA values of the top five influencing factors were instrumental activities of daily living ability (123.18), walking ability (92.71), activities of daily living ability (61.37), balance test performance (40.92), and age (40.51) (Figure 2).

#### 3.1.3 Random forest analysis

**FIGURE 3:**
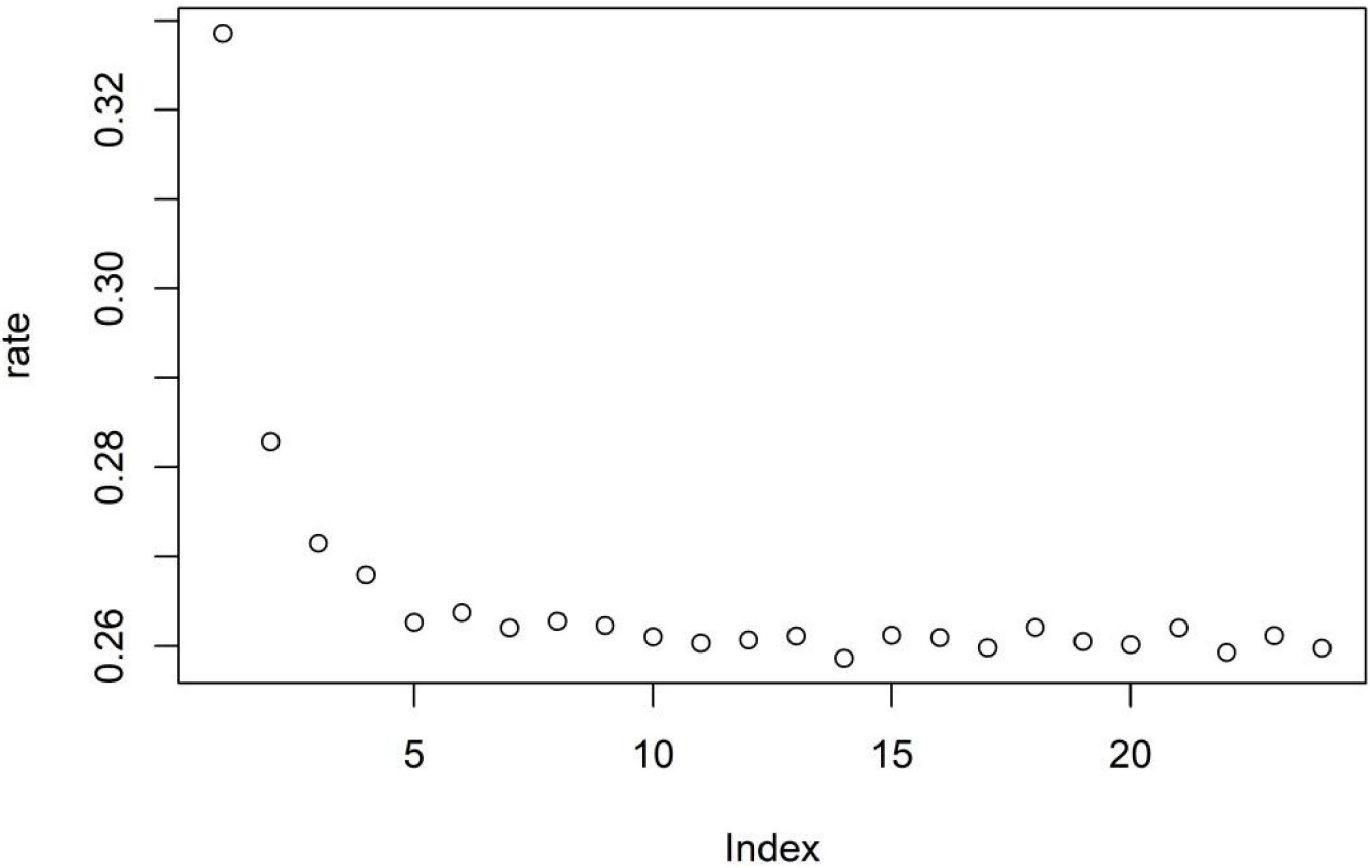
Random forest out-of-bag error rate.

On the basis of the results of the importance ranking of the variables, a stepwise random forest analysis was performed using the variables with the highest importance to find the smallest error rate and filter the number of variables for the purpose of dimensionality reduction. According to the results shown in Figure 3, the lowest out-of-bag error rate of 25.86% was found when the number of variables was 14. This shows that the selection of variables with an importance ranking in the top 14 can achieve high importance and a low error rate in the data analysis. The variables ranked in the top 14 in terms of importance were instrumental activities of daily living ability (123.18), walking test performance (92.71), activities of daily living ability (61.37), balance test performance (40.92), age (40.51), participation in social activities (35.89), BMI (35.69), literacy level (30.93), gait speed (30.49), sit-to-stand test performance (30.17), ethnicity (27.85), main source of income (21.12), current employment status (20.52), and whether they exercised (19.90).

#### 3.1.4 Multifactor logistic regression analysis model

A multifactorial logistic regression analysis was conducted with the top 14 influences screened for importance by the random forest algorithm as the independent variables and the occurrence of cognitive impairment as the dependent variable. The variables and their assigned values are shown in Supplementary Table 2. The results of the analyses revealed that instrumental activities of daily living ability, walking ability, activities of daily living ability, age, participation in social activities, BMI, literacy, gait speed, sit-to-stand test performance, main economic source, current employment status, and exercise had an impact on the cognitive impairment of elderly individuals (Table 2).

**TABLE 2:** Multivariate logistic regression analysis model.

Variable	Normal (*N* = 9,266)	Cognitive impairment (*N* = 3,066)	OR (95% CI)	*P*
Instrumental activities of daily living ability	19.6 ± 5.2	12.5 ± 7.3	0.89 (0.88–0.90)	< 0.001
Walking test	24.1 ± 8.6	15.3 ± 10.8	0.97 (0.96–0.98)	< 0.001
Activities of daily living	92.4 ± 15.7	72.2 ± 29.4	0.99 (0.99–1.00)	< 0.001
Balance test	2.8 ± 1.7	1.6 ± 1.8	0.97 (0.93–1.00)	0.063
Age	69.1 ± 8.0	73.7 ± 8.9	1.02 (1.02–1.03)	< 0.001
**Participate in social activities**				
No (reference)	3,833 (41.4%)	1,738 (56.7%)	–	–
1–3 days per week	3,156 (34.1%)	826 (26.9%)	0.69 (0.61–0.78)	< 0.001
4–6 days per week	1,263 (13.6%)	294 (9.6%)	0.80 (0.67–0.96)	0.018
Everyday	1,014 (10.9%)	208 (6.8%)	0.59 (0.49–0.71)	< 0.001
BMI	24.1 ± 3.7	24.9 ± 3.8	1.08 (1.07–1.10)	< 0.001
**Educational level**				
Illiteracy (reference)	68 (0.7%)	20 (0.7%)	–	–
Primary and below	4,506 (48.6%)	1,681 (54.8%)	1.69 (0.93–3.08)	0.086
Junior high school	2,288 (24.7%)	884 (28.8%)	3.57 (1.95–6.53)	< 0.001
High school or secondary school	1,558 (16.8%)	334 (10.9%)	2.11 (1.14–3.89)	0.017
University or college	631 (6.8%)	113 (3.7%)	2.02 (1.06–3.83)	0.032
Undergraduate and above	215 (2.3%)	34 (1.1%)	1.53 (0.73–3.20)	0.255
Gait speed	1.4 ± 1.4	0.9 ± 1.2	1.16 (1.11–1.21)	< 0.001
Sit-to-stand test	1.7 ± 1.7	0.8 ± 1.4	0.88 (0.85–0.92)	< 0.001
**Ethnic groups**				
Han Chinese (reference)	6,125 (66.1%)	1,782 (58.1%)	–	–
National minority	3,141 (33.9%)	1,284 (41.9%)	1.00 (0.89–1.11)	0.954
**Main economic sources**				
Retirement salary or pension (reference)	6,397 (69%)	1,843 (60.1%)	–	–
From children	1,094 (11.8%)	650 (21.2%)	1.53 (1.33–1.77)	< 0.001
Income from labour	1,128 (12.2%)	293 (9.6%)	1.23 (1.04–1.46)	0.018
Without financial resources	647 (7%)	280 (9.1%)	0.93 (0.77–1.13)	0.461
**Current employment status**				
Retirement (reference)	8,289 (89.5%)	2,747 (89.6%)	–	–
Incumbency	315 (3.4%)	55 (1.8%)	1.02 (0.73–1.44)	0.893
Farmer	227 (2.4%)	73 (2.4%)	0.99 (0.72–1.36)	0.943
Professional	217 (2.3%)	112 (3.7%)	1.54 (1.16–2.05)	0.003
Unemployed	218 (2.4%)	79 (2.6%)	0.90 (0.66–1.23)	0.513
**Exercise**				
No (reference)	2,532 (27.3%)	1,328 (43.3%)	–	–
1∼3 days per week	2,914 (31.4%)	850 (27.7%)	1.31 (1.14–1.50)	< 0.001
4–6 days per week	1,268 (13.7%)	305 (9.9%)	1.17 (0.97–1.42)	0.099
Everyday	2,552 (27.5%)	583 (19%)	1.30 (1.13–1.51)	< 0.001

#### 3.1.5 Model performance

**FIGURE 4:**
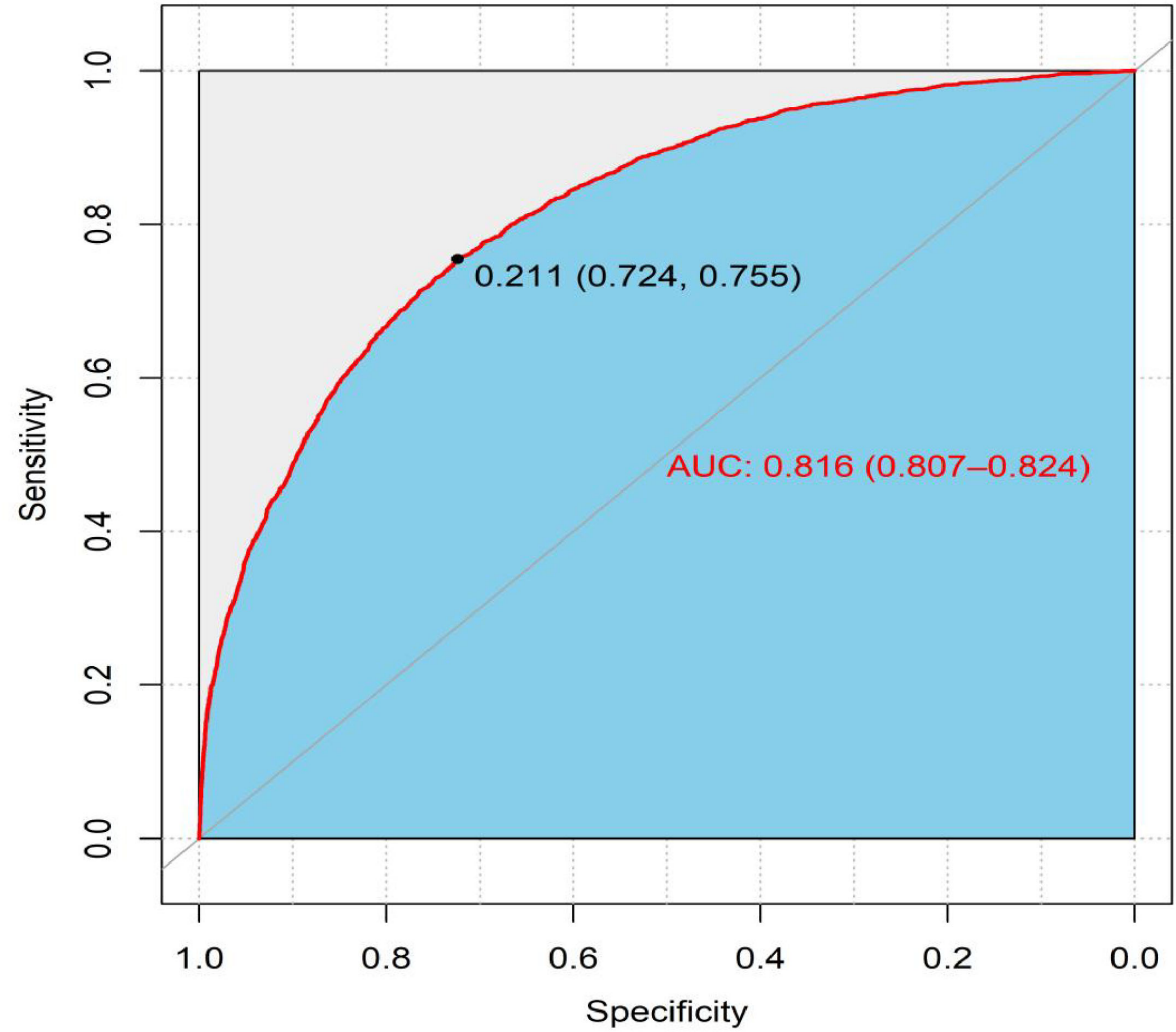
Receiver operating characteristic (ROC) curves for the predictive model of cognitive impairment.

The model established with the above 12 independent predictors reached an area under the curve of 0.816 (95% CI: 0.807∼0.824); the risk prediction value of 0.211 was the best cut-off value and showed good sensitivity (75.50%), specificity (72.40%), accuracy (73.14%), F1 score (0.802), precision (89.91%), and recall (72.38%) (Figure 4).

#### 3.1.6 Construction of the nomogram

**FIGURE 5:**
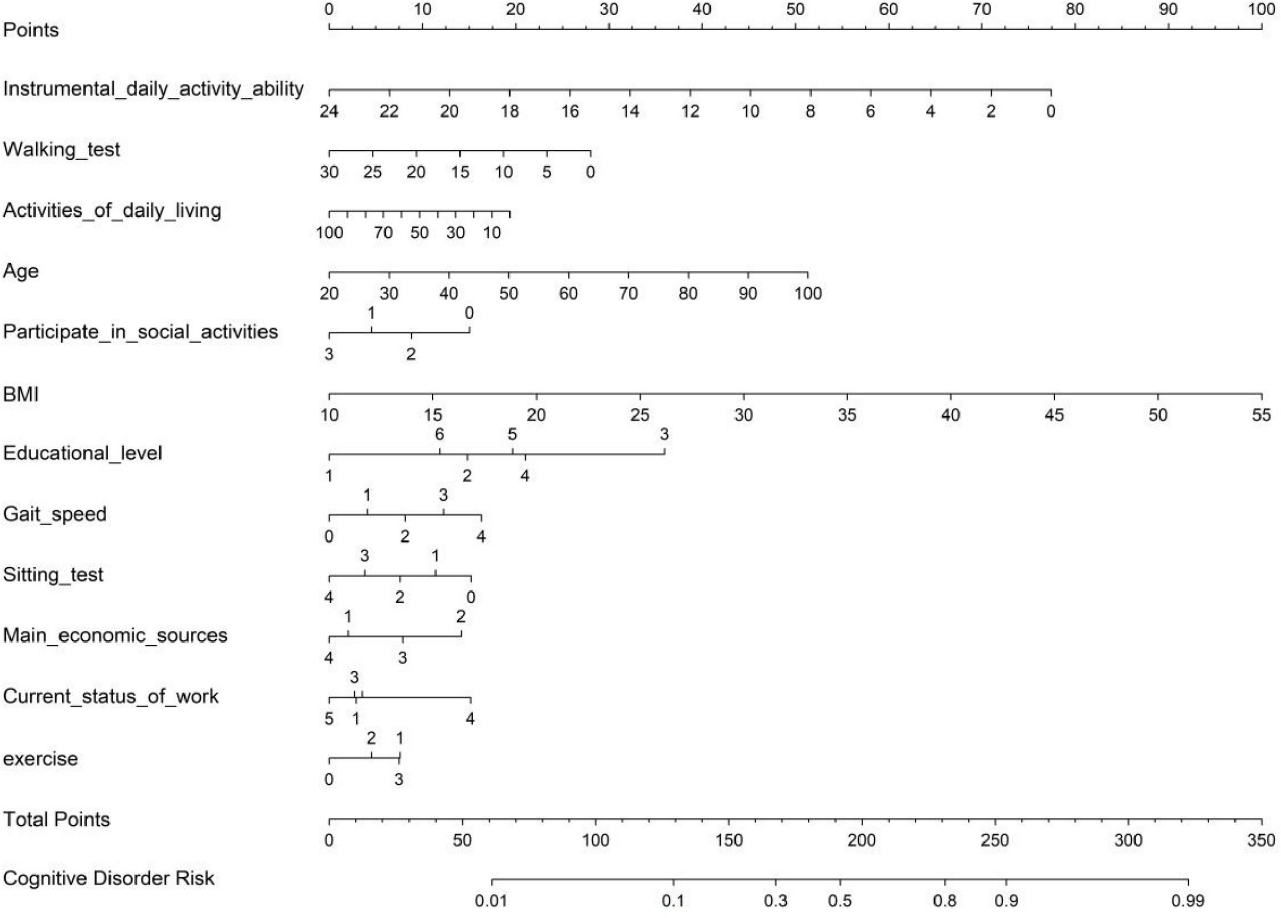
Nomogram of the predictive model for cognitive impairment in elderly patients.

With R software, the 12 significant influencing factors were used to construct a nomogram (Figure 5). For the elderly patients, each indicator was projected upwards to a small scale (points) to obtain a score for each item. The total score was obtained by summing the scores, and the higher the total score was, the greater the likelihood of cognitive impairment.

### 3.2 Discussion

#### 3.2.1 Detection rate of cognitive impairment among elderly people in Northwest China

The results of this study revealed that the detection rate of cognitive impairment in older adults in northwestern China was 24.86%, which was higher than that reported in a cross-sectional study investigating the prevalence of cognitive impairment in 3,768 older adults aged 60 years and older in northern and northwestern China (22.24%) (Qin et al., 2022). An in-depth study revealed that the prevalence of cognitive impairment in older adults in southwestern and northwestern China was 1.81 times greater than that in those in the northern region (Qin et al., 2022). These findings are also higher than the results of a cross-sectional study on the prevalence of cognitive impairment among 2,598 older Han Chinese adults included in three villages and six communities in Liuyang city, Hunan Province, China (21.48%) (Xu et al., 2023). A meta-analysis reported that the prevalence of dementia was greater in western China (14.33%) than in eastern China (13.41%) (Xue et al., 2018). These results and the analyses in this study revealed that the prevalence of cognitive impairment in older Chinese adults was strongly associated with geographic location, with the highest prevalence of cognitive impairment occurring in older adults in the northwestern part of the country. Dietary habits may be the main reason for this difference. Northwest China, as a multiethnic area, has a variety of dietary habits and favors spicy, sweet and salty diets, which often lead to hypertension, diabetes mellitus, and chronic kidney disease (Zhou et al., 2021). A previous study by our group revealed that chronic disease is an important factor involved in the occurrence of cognitive impairment. Dietary habits play an important role in the geographical factors of cognitive impairment. In addition, economic development, education, and medical care in different regions may also be responsible for such differences (Wang et al., 2024).

#### 3.2.2 Random forest algorithm and multifactorial logistic regression analysis predictive model of cognitive impairment among older adults in Northwest China

This study used a random forest algorithm and multifactorial logistic regression analysis model to analyze the factors influencing cognitive impairment in older adults. The results of this study revealed that the risk of cognitive impairment was 1.54 times greater (95% CI: 1.16–2.05) in older adults whose current work situation was freelance compared with retired older adults. In addition, the risk of cognitive impairment in older adults whose main source of income was from their children’s contributions or from their own labor was 1.53 times greater than that in older adults who were retired and 1.23 times greater than that in older adults who were receiving a pension. This type of freelance work, which is often associated with unstable income and thus a greater dependence on the basic subsistence allowance as a source of income, is associated with a greater risk of cognitive impairment in older adults with these conditions (Wang et al., 2020). As noted in the present study, it is possible that financial insecurity and income imbalance places older adults at greater risk of developing anxiety and restlessness, which may have an impact on the development of cognitive impairment.

The present results showed that the ability to perform instrumental activities of daily living was a protective factor against cognitive impairment in older adults (OR = 0.89, 95% CI: 0.88–0.90). Lahav and Katz (2020) reported that higher IADL scores indicate greater cognitive levels and a lower incidence of cognitive impairment in older adults. Mild changes in IADL can predict future cognitive decline, where the ability to manage finances may be one of the earliest IADL changes in dementia (Gold, 2012). In addition, one study revealed a strong association between walking function and cognitive function, as well as an important measure of physical function and health in older adults (O’Brien and Holtzer, 2021). The results of this study revealed that older adults with lower scores on the walking test were more likely to have cognitive impairment (OR = 0.97, 95% CI: 0.96–0.98). Therefore, by monitoring and assessing changes in walking ability in older adults, the risk of cognitive impairment can be detected early, providing a basis for early intervention and treatment. Importantly, the relationship between walking ability and cognitive impairment in older adults is complex, and the exact causal relationship remains incompletely understood (Lam et al., 2018). Therefore, further studies are still needed to better understand the relationship between gait speed and cognitive impairment in elderly people and to provide more targeted approaches and measures for the prevention and intervention of cognitive impairment.

Finally, similar to the findings of many previous studies (Marioni et al., 2015; Park et al., 2017), the more types or greater frequency of social activities such as dating, mahjong, and dancing that an individual reported in this study, the greater the likelihood that the cognitive functioning trajectory was associated with either the “medium stability group” or the “high stability group.” When older people have fewer social networks and less contact with family, neighbors or friends, they are more likely to be associated with low- and medium-stability groups over time. Social activities help stimulate the brain and motivate older adults to maintain active cognitive functioning. Through social activities such as interacting with others, participating in group activities, and sharing interests, older adults can exercise their memory, attention, and logical thinking skills (Lee et al., 2018). In addition, social activities help reduce loneliness and cognitively impaired emotions and promote physical and mental health (Langa and Levine, 2014). Therefore, managers in government departments should provide a safe, friendly, and supportive socializing environment to create more opportunities for older people to socialize and participate, which can help to promote their mental health and cognitive abilities.

#### 3.2.3 The nomogram model

Nomograms can create simple graphical representations of statistical prediction models that generate numerical probabilities of clinical events without the need for electronic devices, with simple addition and a user-friendly, practical interface. In this study, a fully integrated visual nomogram for predicting cognitive impairment risk in elderly individuals was developed, which can be useful for providing early individualized predictive risk probability estimates for older adults. The validity, discrimination and clinical usefulness of the model were estimated, and the results showed that the predictive model was well fitted. In this study, a total score of 24 (secondary and above education level), 20 (primary education level) or 17 (illiterate) for cognitive impairment on the Mini-Mental State Examination (MMSE) was used as a cut-off value for cognitive impairment to construct a predictive model of risk for cognitive impairment in older adults. Currently, the effectiveness of interventions for preventing cognitive impairment is less clear (Huang et al., 2022), prompting us to construct a prediction model for the risk of cognitive impairment in older adults. By accurately predicting the degree of model fit, the models included the ability to perform instrumental activities of daily living, participation in social activities, literacy level, gait speed, sit-to-stand test, primary economic source, and current work situation to arrive at a sufficiently predictive nature. This is because the nomogram clearly shows how different age groups, sexes and other groups compare in terms of the risk of cognitive impairment, making the data more visualizable. In addition, nomograms can quickly reveal whether the risk of cognitive impairment increases with age, helping decision-makers better understand the potential risks. As a result, health care professionals are able to predict the risk of cognitive impairment in older adults in a more streamlined manner by obtaining patient information.

#### 3.2.4 Limitations and implications

There are several limitations in this study. (1) The evaluation of patients with cognitive impairment was based on a commonly used neuropsychology test, and its diagnostic performance for cognitive impairment was limited. The diagnosis of cognitive impairment should be based on comprehensive clinical evaluation or proof in future research. (2) The predictive utility of the composite risk score for cognitive impairment may vary across countries and regions because of the influence of genetics, demographics, and economics on the aging process. Thus, more large-scale and well-designed studies are necessary to replicate our analyses in other regions of Northwest China to evaluate the validity of the composite risk score. (3) The cross-sectional design of our study means that the temporal relationship between the predictors and the outcome cannot be established, and there may be reverse causation or confounding effects that have not been accounted for. A cohort study is needed to confirm the predictive value of the model and improve it. The predictive value of the model needs to be confirmed and enhanced by cohort studies. (4) Our predictive model lacked external evaluation, which needs to be verified in the future. (5) The model did not contain information on genetic biomarkers or environmental factors. However, an increasing number of studies have shown that genes such as *ADAMTS9*, *APOE*, *BDNF*, *CASS4*, *COMT*, *CR1*, *DNMT3A*, *REST*, and *TOMM40* are significantly correlated with cognitive impairment and have the potential to enhance the ability of models to predict cognitive decline (Lin et al., 2017; Varatharajah et al., 2019). The predictive performance would likely increase significantly in future studies if genetic and environmental biomarker data were incorporated into analyses. However, the most prominent strengths of this study include the large sample size. The present study can be used to assess the risk of cognitive impairment as well as for the development of preventive interventions targeting variable risk factors, with good guiding implications in public health and clinical settings.

## 4 Conclusion

In this study, we noted a high prevalence of cognitive impairment among older adults in Northwest China. The combination of machine learning and logistic regression yielded a practical cognitive impairment prediction model, which included 12 predictive factors and achieved good performance. On the basis of the results of the nomogram, early intervention can be conducted in high-risk groups to prevent or delay further development of cognitive impairment. In the future, more large-scale and well-designed studies are necessary to replicate our analyses in other regions of Northwest China to evaluate the validity of the composite risk score.

## Data Availability

The original contributions presented in this study are included in this article/Supplementary material, further inquiries can be directed to the corresponding authors.
