# Supplementary material for: Construction of a predictive model for cognitive impairment among older adults in Northwest China
Source: Front Aging Neurosci. 2025 Jul 31;17:1487838. doi: 10.3389/fnagi.2025.1487838 (PMC12350356; doi:10.3389/fnagi.2025.1487838)
Supplement: Supplementary file 1 [file Table_1.docx]

| **Supplementary Text 1. Comparison of the occurrence of cognitive dysfunction in older people with different characteristics** | | | |
| --- | --- | --- | --- |
| variable | cognitive normal | cognitive impairment | *P* value |
|  | (N=9266) | (N=3066) |  |
| **sex** |  |  |  |
| male | 4547 (49.1%) | 1447 (47.2%) | 0.0748 |
| women | 4719 (50.9%) | 1619 (52.8%) |  |
| **BMI** |  |  |  |
| Mean (SD) | 24.1 (3.66) | 24.9 (3.82) | <0.001 |
| **age** |  |  |  |
| Mean (SD) | 69.1 (7.98) | 73.7 (8.85) | <0.001 |
| **nationality** |  |  |  |
| han ethnicity | 6125 (66.1%) | 1782 (58.1%) | <0.001 |
| national minority | 3141 (33.9%) | 1284 (41.9 %) |  |
| **educational attainment** |  |  |  |
| illiteracy | 68 (0.7%) | 20 (0.7%) | <0.001 |
| Primary and below | 4506 (48.6%) | 1681 (54.8%) |  |
| junior high school | 2288 (24.7%) | 884 (28.8%) |  |
| High school or secondary school | 1558 (16.8%) | 334 (10.9 %) |  |
| University or college | 631 (6.8%) | 113 (3.7 %) |  |
| Undergraduate and above | 215 (2.3%) | 34 (1.1%) |  |
| **marital status** |  |  |  |
| unmarried | 27 (0.3%) | 22 (0.7 %) | <0.001 |
| married | 8140 (87.8%) | 2329 (76.0 %) |  |
| widowed | 929 (10.0%) | 665 (21.7 %) |  |
| divorced | 115 (1.2%) | 36 (1.2 %) |  |
| other | 55 (0.6%) | 14 (0.5%) |  |
| **Type of residence** |  |  |  |
| municipalities | 6740 (72.7%) | 2045 (66.7 %) | <0.001 |
| countryside | 2526 (27.3%) | 1021 (33.3 %) |  |
| **Residence** |  |  |  |
| Living with family | 8763 (94.6%) | 2836 (92.5 %) | <0.001 |
| live alone | 474 (5.1%) | 164 (5.3 %) |  |
| Institutions for elderly individuals | 29 (0.3%) | 66 (2.2 %) |  |
| **Current work status** |  |  |  |
| retirement | 8289 (89.5%) | 2747 (89.6 %) | <0.001 |
| Incumbency | 315 (3.4%) | 55 (1.8%) |  |
| farmer | 227 (2.4%) | 73 (2.4 %) |  |
| Professional | 217 (2.3%) | 112 (3.7 %) |  |
| unemployed | 218 (2.4%) | 79 (2.6 %) |  |
| **Type of medical insurance** |  |  |  |
| urban workers | 4399 (47.5%) | 1096 (35.7 %) | <0.001 |
| urban dwellers | 3255 (35.1%) | 1290 (42.1%) |  |
| New Agricultural Cooperative Society (NACS) | 1203 (13.0%) | 505 (16.5 %) |  |
| other | 409 (4.4%) | 175 (5.7 %) |  |
| **Primary financial resources** |  |  |  |
| Retirement pay or pension | 6397 (69.0%) | 1843 (60.1%) | <0.001 |
| From children | 1094 (11.8%) | 650 (21.2 %) |  |
| income from labour | 1128 (12.2%) | 293 (9.6 %) |  |
| without financial resources | 647 (7.0%) | 280 (9.1%) |  |
| **Presence of chronic diseases** |  |  |  |
| no | 2348 (25.3%) | 499 (16.3 %) | <0.001 |
| yes | 6918 (74.7%) | 2567 (83.7 %) |  |
| **Whether or not the drug is used for a long period of time** | | | |
| no | 3393 (36.6%) | 853 (27.8%) | <0.001 |
| yes | 5873 (63.4%) | 2213 (72.2 %) |  |
| **Alcohol consumption** |  |  |  |
| Never | 6631 (71.6%) | 2226 (72.6 %) | 0.00214 |
| former | 1978 (21.3%) | 678 (22.1%) |  |
| current | 657 (7.1%) | 162 (5.3 %) |  |
| **Smoking** |  |  |  |
| Never | 6949 (75.0%) | 2282 (74.4 %) | <0.001 |
| Former | 1557 (16.8%) | 622 (20.3 %) |  |
| Smoke less than half a pack | 568 (6.1%) | 121 (3.9 %) |  |
| Smoking more than half a pack | 192 (2.1%) | 41 (1.3 %) |  |
| **Whether or not annual medical check-ups are conducted** | | | |
| no | 2196 (23.7%) | 1009 (32.9 %) | <0.001 |
| yes | 7070 (76.3%) | 2057 (67.1%) |  |
| **Participation in social activities** |  |  |  |
| none | 3833 (41.4%) | 1738 (56.7 %) | <0.001 |
| 1~3 days per week | 3156 (34.1%) | 826 (26.9 %) |  |
| 4-6 days per week | 1263 (13.6%) | 294 (9.6 %) |  |
| everyday | 1014 (10.9%) | 208 (6.8%) |  |
| **Whether or not you exercise** |  |  |  |
| none | 2532 (27.3%) | 1328 (43.3 %) | <0.001 |
| 1~3 days per week | 2914 (31.4%) | 850 (27.7 %) |  |
| 4-6 days per week | 1268 (13.7%) | 305 (9.9 %) |  |
| everyday | 2552 (27.5%) | 583 (19.0 %) |  |
| **Social support** |  |  |  |
| none | 368 (4.0%) | 237 (7.7%) | <0.001 |
| Material support only | 235 (2.5%) | 160 (5.2 %) |  |
| Emotional support only | 283 (3.1%) | 140 (4.6%) |  |
| Adequate material and emotional support | 8380 (90.4%) | 2529 (82.5 %) |  |
| **Mini-Mental State Examination (MMSE) score** | | | |
| Mean (SD) | 27.6 (2.62) | 17.1 (5.77) | <0.001 |
| **MMSE-directionality** |  |  |  |
| Mean (SD) | 9.72 (0.656) | 7.08 (2.60) | <0.001 |
| **MMSE-ability to remember** |  |  |  |
| Mean (SD) | 2.86 (0.397) | 1.88 (1.03) | <0.001 |
| **MMSE-attention** |  |  |  |
| Mean (SD) | 4.07 (1.32) | 1.53 (1.30) | <0.001 |
| **MMSE-recall ability** |  |  |  |
| Mean (SD) | 2.71 (0.575) | 1.38 (0.966) | <0.001 |
| **MMSE-verbal ability** |  |  |  |
| Mean (SD) | 2.94 (0.284) | 2.26 (0.984) | <0.001 |
| **MMSE-three-step command** |  |  |  |
| Mean (SD) | 5.35 (0.952) | 2.96 (1.68) | <0.001 |
| **Balanced Test Assessment Total Score** | | | |
| Mean (SD) | 2.80 (1.67) | 1.62 (1.77) | <0.001 |
| **Total Gait Speed Assessment Score** | | | |
| Mean (SD) | 1.42 (1.38) | 0.946 (1.21) | <0.001 |
| **Total score for sitting and standing test assessment** | | | |
| Mean (SD) | 1.71 (1.74) | 0.776 (1.36) | <0.001 |
| **Total Walking Test Assessment Score** | | | |
| Mean (SD) | 24.1 (8.63) | 15.3 (10.8) | <0.001 |
| **Total score for instrumental daily activity skills** | | | |
| Mean (SD) | 19.6 (5.20) | 12.5 (7.34) | <0.001 |
| **Total score for ability to perform activities of daily living** | | | |
| Mean (SD) | 92.4 (15.7) | 72.2 (29.4) | <0.001 |
