# Supplementary material for: Construction of a predictive model for cognitive impairment among older adults in Northwest China
Source: Front Aging Neurosci. 2025 Jul 31;17:1487838. doi: 10.3389/fnagi.2025.1487838 (PMC12350356; doi:10.3389/fnagi.2025.1487838)
Supplement: Supplementary file 2 [file Table_2.docx]

| Variable assignment table | |
| --- | --- |
| Influencing factor | Assignment situation |
| age | carry over the original value |
| BMI | carry over the original value |
| Instrumental Activities of Daily Living ability | carry over the original value |
| Walking test | carry over the original value |
| Activities of Daily Living (ADLs) | carry over the original value |
| Balance test | carry over the original value |
| gait speed | carry over the original value |
| Sit-to-stand test | carry over the original value |
| Nationality | 1 = Han Chinese, 2 = ethnic minorities |
| Participation in social activities | 1=No, 2=1 to 3 days per week, 3=4 to 6 days per week, 4=daily |
| Main economic sources | 1=retired salary or pension, 2=given by children, 3=earned from labour, 4=no financial resources |
| Current employment status | 1=retired, 2=working, 3=farmer, 4=freelance, 5=no work |
| Exercise status | 1=No, 2=1 to 3 days per week, 3=4 to 6 days per week, 4=daily |
| educational attainment | 1 = illiterate, 2 = primary school and below, 3 = middle school, 4 = high school or secondary school, 5 = university or college, 6 = bachelor's degree and above |
